# A spatial perspective on flowering in cauliflorous cacao: architecture defines flower cushion location, not its early activity

**DOI:** 10.1093/aob/mcaf107

**Published:** 2025-06-10

**Authors:** Thomas Wibaux, Pierre-Éric Lauri, Antoine Alban M’Bo Kacou, Olsen Pondo Kouakou, Rémi Vezy

**Affiliations:** CIRAD, UMR ABSys, Montpellier F-34398, France; ABSys, CIHEAM, CIRAD, INRAE, Institut Agro, Université de Montpellier, Montpellier, France; CEA-CCBAD, Université Félix Houphouët-Boigny, 22 BP 463 Abidjan 22, Côte d’Ivoire; ABSys, CIHEAM, CIRAD, INRAE, Institut Agro, Université de Montpellier, Montpellier, France; CEA-CCBAD, Université Félix Houphouët-Boigny, 22 BP 463 Abidjan 22, Côte d’Ivoire; CIRAD, UMR ABSys, Montpellier F-34398, France; CIRAD, UMR AMAP, Montpellier F-34398, France; AMAP, CIRAD, CNRS, INRAE, IRD, Université de Montpellier, Montpellier, France

**Keywords:** *Theobroma cacao* L, cauliflory, plant architecture, axis geometry, floral bud, flower cushion, sciaphilous species, shade effect

## Abstract

**Background and Aims:**

*Theobroma cacao* L., a tropical sciaphilous tree, exhibits cauliflory, with persistent flowering sites known as flower cushions. Cushions develop from floral buds located at the axils of leaves or cataphylls. They can sustain recurrent flowering and contribute to the fruit production of the tree throughout its lifespan. However, factors influencing their formation and flowering activity remain poorly understood.

**Methods:**

Architectural and geometrical measurements, combined with weekly monitoring of flowering activity at the node scale, were conducted on plagiotropic branches of two cacao genotypes under medium and heavy shade. We investigated how architecture and geometry influence cushion formation, frequency and duration of flowering episodes, and synchrony of flowering among cushions at different scales.

**Key Results:**

Flower cushions developed once a flush (growth unit) had reached a specific ontogenetic age, defined by its position along the shoot (axis). The probability of flower cushion formation was then determined primarily by the position of the node (phytomer) within the flush and its basal diameter. Heavy shade (90 % light reduction) greatly limited cushion formation, regardless of the architectural traits or growth characteristics of the node, flush or shoot. In contrast, the temporal activity of the flower cushions was not related to architectural or geometrical factors, and flowering occurred with moderate synchrony at the growth unit, axis and branch scales.

**Conclusions:**

These findings indicate the dual importance of shoot ontogeny and node-specific traits in flower cushion formation in this cauliflorous species. They also demonstrate the overall negative influence of heavy shade on flower cushion development, and the absence of architectural constraints on the flowering activity of cushions during the first reproductive phase of the tree. Further analyses are needed to gain a better understanding of the hormonal and carbohydrate regulation of flowering and fruiting in productive trees of this cauliflorous species.

## INTRODUCTION

Cauliflory refers to the development of flowers and fruits on the lower parts of trees, directly on the trunks, branches and woody twigs (Endress, 2010). It is a common reproductive strategy among tropical understorey species, offering several advantages, particularly by providing easier access to pollinators and dispersers (Warren *et al.*, 1997; Fonseca and Lohmann, 2017). Cauliflory exhibits various forms, with flowering limited to the trunk (trunciflory) or extending to both the trunk and main branches (ramiflory), whether leafless or leafy (Endress, 2010; Quintanar *et al.*, 2023). Cauliflorous inflorescences can sometimes develop adventitiously, but they typically originate from axillary buds at node level formed during stem elongation, as for most plants (Bell and Bryan, 1991; Endress, 2010). However, cauliflorous species exhibit two key differences from typical plants in their flowering mechanisms. First, the buds can remain suppressed, maintaining minimal growth until conditions are favourable for a primary inflorescence to emerge. This delay can extend to several years, allowing inflorescences to develop even on the oldest, basal parts of the tree (Corner, 1949; Bell and Bryan, 1991). Second, new buds develop at the base of dehiscent flowers, facilitating repeated flowering at the same floral site. This adaptation enables sustained flowering on ageing nodes along the trunk or woody branches (Owens *et al.*, 1995; Endress, 2010; Fonseca and Lohmann, 2017). The sites of recurrent flowering typically retain the ability to produce flowers throughout the life of the tree and are commonly referred to as ‘flower cushions’ (Lent, 1966; Leal and Paull, 2023).

The cacao tree (*Theobroma cacao* L.), a species of major agricultural importance, is native from the understorey of Amazon forests and exhibits both cauliflory and ramiflory. The tree follows Nozeran’s architectural model, which is characterized by a dimorphic branching system (Hallé, 1961; Hallé *et al.*, 1978). An orthotropic shoot with determinate growth and spiral phyllotaxy forms the trunk. After ∼1–3 years, vertical growth ceases when the shoot apex aborts, leading to the development of a whorl of three to six plagiotropic branches, known as the ‘jorquette’, produced by syllepsis from the distal axillary buds. These primary plagiotropic branches exhibit distichous phyllotaxy, arranged in dorsiventral symmetry, and can produce further plagiotropic branches via prolepsis from axillary buds, each maintaining similar morphology. Growth is rhythmic, and each flush typically produces a succession of 0–10 cataphylls, followed by 2–12 developed leaves (Greathouse and Laetsch, 1969; Vogel, 1975). The tree resumes vertical growth, or reiterates from its base, by emitting new orthotropic axes reproducing the same dimorphic architectural model (Greathouse and Laetsch, 1969; Hallé *et al.*, 1978; Wheat, 1979).

Cultivated cacao is still primarily grown from seedlings, with trees typically maintained on one or two stems and kept short by pruning orthotropic shoots (Tosto *et al.*, 2023). Vegetative propagation via grafting or budding is also used, albeit less frequently. When propagated from plagiotropic shoots, the characteristic plagiotropy and associated architecture largely persists, with more or less upward reorientation of growth (i.e. ‘topophysis’; Hallé *et al.*, 1978; Bell and Bryan, 1991; Bartley, 2005).

Both orthotropic and plagiotropic axes bear nodes that carry either a developed leaf or a cataphyll. Each node also carries two vertically arranged axillary buds: an upper principal (vegetative) bud and a subordinate bud that is almost exclusively dedicated to flowering (Wheat, 1979). The subordinate bud is located in a depression beneath the principal vegetative axillary bud and above the base of the subtending leaf, leaf scar or cataphyll, following the phyllotaxy of the axis (Lent, 1966; Greathouse and Laetsch, 1969; Wheat, 1979). It can remain dormant for extended periods, sustaining minimal growth until dormancy is released (Lent, 1966; Wheat, 1979; Bell and Bryan, 1991; Swanson *et al.*, 2008).

**Fig. 1.**
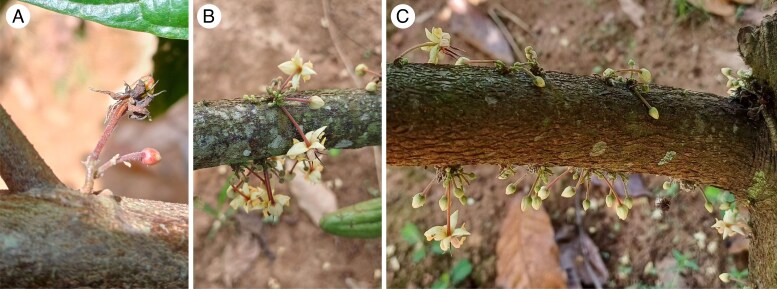
Flowers of *Theobroma cacao* L.: (A) unopened and recently pollinated flowers, borne on a visible cyme, located between a vegetative ramification and a leaf scar; (B) flower cushions bearing flower buds, growing flowers and flowers at anthesis; and (C) flower cushions along an axis, following the phyllotaxy.

The inflorescence of the cacao tree is a cyme with short branches (<1 mm). Its base is usually hidden under the bark, and the flower pedicel is long and arises from the bark (Fig. 1A). As multiple inflorescences develop successively at the same position, they form clusters of compressed cymes that ultimately give rise to the characteristic swelling called a ‘flower cushion’ (Alvim, 1984; Lachenaud, 1991; Swanson *et al.*, 2008; Fig. 1B, C). When cacao is grown from a seed, flowering usually begins after 2–4 years in a sudden transition initiated by the establishment of the ‘jorquette’ (Swanson *et al.*, 2008; Carr and Lockwood, 2011). During this initial flowering stage, inflorescences develop at the axils of the oldest nodes on the orthotropic trunk, where subordinate buds had remained suppressed for up to 4 years. Gradually, as the plagiotropic axes elongate and branch, inflorescences also develop along the primary branches and higher-order ramifications. In contrast, cacao propagated from mature plagiotropic axes flowers more quickly (Bartley, 2005). Carr and Lockwood (2011) report that clones of budded plagiotropic axes can begin flowering as soon as the second flush has hardened.

Despite the global importance of cacao, the conditions that trigger flower bud activation and initial flowering remain poorly understood. It is also unclear which nodes gain flowering capacity and in what architectural, physiological or growth conditions. Some authors suggest that a branch may bear flower when it has lignified (Bartley, 2005) or has reached >1 cm in diameter (Alvim, 1984). Although the literature on factors influencing the formation of floral cushions is limited, more attention has been paid to the temporal dynamics of flowering. Most studies focused on flowering timing and intensity at the whole-tree scale, typically monitoring portions of the trunk and/or branches and assuming synchronous, homogeneous activity of flower cushions (Alvim, 1966; Boyer, 1970; Asomaning *et al.*, 1971; Ampofo and Bonaparte, 1979; Mossu *et al.*, 1981; Young, 1984; Valle *et al.*, 1990; Tosto *et al.*, 2022). A few studies have used finer scales, distinguishing between the orthotropic trunk(s) and plagiotropic branches, yet still assumed a degree of homogeneity in the temporal activity across flower cushions at those scales (Omolaja *et al.*, 2009; Adjaloo *et al.*, 2012; Wibaux *et al.*, 2024). Notably, each of these studies reported differences in flowering timing, intensity and duration among different parts of the tree. Finally, only a small set of controlled-environment experiments from 1965 conducted in a growth chamber addressed the temporal variations in the activity of individual flower cushions (Sale, 1969, 1970*a*, *b*), yet even these were ultimately summarized at the tree scale. Consequently, there is a significant gap in our understanding of flowering dynamics at the scale of the flower cushion, but also in terms of how flowering activity is coordinated (or not) across axes and branches. A clearer understanding of how the architectural and growth traits influence both the spatial positioning and the flowering activity of cushions would be invaluable for refining agronomic practices and breeding strategies in cacao.

This study examines the effects of shade on cacao architecture, growth and flowering by monitoring the formation and activity of flower cushions at the node scale on plagiotropic branches of young clones of grafted cacao trees. Our objectives were 3-fold: (1) to identify the factors influencing flower cushion formation at the phytomer scale; (2) to describe the temporal dynamic of flower cushion activity and determine which factors govern the recurrence of flowering and the duration of subsequent flowering episodes; and (3) to assess the synchrony of flowering activity among cushions within growth units, axes and branches.

## MATERIALS AND METHODS

A glossary of specialized terms used in this article, in reference to cacao architectural and morphological features, is presented in Table 1.

**Table 1.** Glossary of specialized terms.

Term	Definition
Phytomer	The basic structural unit of the plant body, consisting of a node, its attached leaf (or leaves), its attached axillary bud(s) and the internode below.^1^
Axis	The main structural framework of a plant, formed by the repeated stacking of phytomers. Each axis has a single insertion point and a single apical meristem.^1^
Branch	The entire structure formed by an aerial axis of branching order *n* and all its successive ramifications of orders *n* + 1, *n* + 2, and so on.^1^
Growth unit	A segment of an axis produced during a single elongation event (flush) of rhythmic growth, typically consisting of a series of phytomers formed without interruption.^1^
Ontogenetic age	The developmental age of a plant organ or structure, based on its position within the developmental cycle. Different organs (axis, growth unit, phytomer) on the same plant can have different ontogenetic ages, even if they formed during the same season.^1^
Othotropic axis	A vertically growing axis that maintains an upright orientation; in cacao, it forms the trunk, and it is characterized by determinate growth and spiral phyllotaxy.^2^
Plagiotropic axis	A horizontally growing axis, forming the branches in the crown of the tree. In cacao, it is associated with lateral spreading growth and distichous phyllotaxy.^2^
Jorquette	In cacao grown from seed, the whorl of three to six primary plagiotropic axes that forms at the top of the orthotropic trunk when its vertical growth terminates.^2^
Syllepsis	The immediate development of a lateral branch from an axillary bud without a period of dormancy; responsible for forming the jorquette branches in cacao.^1^
Prolepsis	The development of a branch from an axillary bud after a period of dormancy; common in higher-order branching in cacao.^1^
Cauliflory	The production of flowers and fruits directly on trunks or older woody branches, rather than on young shoots.^3^
Trunciflory/ramiflory	Forms of cauliflory where flowering is restricted to the trunk(s) (trunciflory) or to older woody branches (ramiflory).^3^
Flower cushion	A swollen, perennial structure formed by the repeated development of inflorescences at the same node on trunks or branches; characteristic of cauliflorous species.^3^
Cataphyll	A reduced, scale-like leaf that often precedes the development of normal foliage leaves during a growth flush.^1^

^1^ Barthélémy and Caraglio (2007). ^2^Hallé *et al.* (1978). ^3^Bell and Bryan (1991) .

### Plant material and experimental design

A study of the effects of shade on cacao growth and reproduction was conducted between 2021 and 2024 on an industrial plantation in central-west Côte d’Ivoire (6°11′18.8″N, 6°07′29.5″W; Wibaux *et al.*, 2025). In June 2021, three hundred 4-month-old cacao seedlings were planted to serve as rootstocks, with a spacing of 2 m along the planting line and 3 m between the rows. The rootstocks were side-grafted in January 2022 with scions collected on plagiotropic shoots of various local genotypes. Both the rootstocks and the locally selected genotypes originated from farmers’ orchards established with uncontrolled germplasm, presumed to have Upper Amazon, Lower Amazon or African Trinitario parentage, as is commonly found in the region (Pokou *et al.*, 2006). The rootstocks were coppiced over the grafting point, and a single axis emerging from the grafted scions was maintained, which, along with its ramifications, was left untrained and unpruned.

**Fig. 2.**
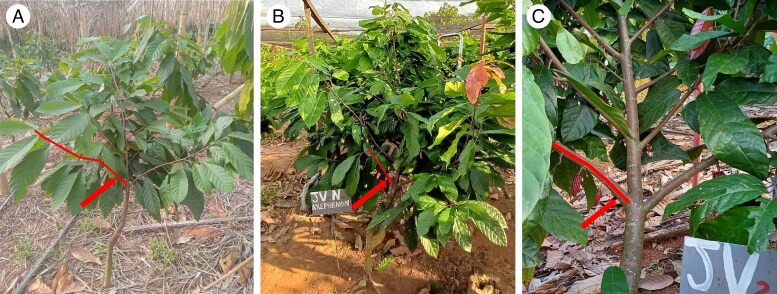
Photographs of: (A) a studied tree and its selected branch (in red) during initial ‘baseline’ architectural measurement campaign ‘MTG_1’; (B) same tree during second architectural measurement campaign ‘MTG_2’ after 10 months of shading treatment, with the base of the selected branch localized in red; (C) zoom on the insertion of the selected branch on the primary axis of the tree.

After 1 year of growth under medium shade [∼60 % of ambient photosynthetically active radiation (PAR); LICOR LI-191R Line Quantum Sensor], two shading treatments were applied in March 2023: ‘medium shade’ (∼60 % of ambient PAR; Aranet PAR Sensor) and ‘heavy shade’ (∼10 % of ambient PAR; Aranet PAR Sensor), using black shading nets placed at 3 m height. Throughout the experiment, supplemental irrigation was provided using micro-sprinklers to maintain a consistent soil water status. Trees were fertilized every 4 months with complete nutrient formulations: urea and triple superphosphate until November 2022, followed by NPK (0-23-18) and urea from February 2023 onwards. The climate and typical crop phenology of the study area are described in detail by Wibaux *et al.* (2024). For this study on flowering, considering the important quantity of data required, we selected nine healthy and well-developed trees of two architecturally contrasted local genotypes, named ‘genotype B’ and ‘genotype V’: two trees of genotype V under heavy shade; two of genotype V under medium shade; two of genotype B under heavy shade and three of genotype B under medium shade. On each tree, a single second-order branch was selected for the study and will be referred to hereafter as the ‘selected branch’ or simply ‘branch’ (Fig. 2).

### Data collection

#### Branch, axis and growth unit architecture and geometry

We conducted a comprehensive architectural description of the ‘selected branch’ from each of the nine cacao trees, at the axis, growth unit and phytomer scales. Data collection involved visual observations and markings on the trees, along with manual measurements of organ dimensions and orientations. The data were recorded as multi-scale tree graphs (MTGs; Godin and Caraglio, 1998) in spreadsheet files and processed using the Julia package *MultiScaleTreeGraph* (Vezy, 2022). This data structure effectively captures both the topology (adjacency relationships between organs) and geometry (organ dimensions) of plant structures across multiple scales by representing their multiscale topological relationships through nested tree graphs (Godin and Caraglio, 1998). An initial baseline architectural measurement campaign (MTG_1) was conducted in January–February 2023, prior to application of the shade treatments, followed by a second campaign (MTG_2) in November–December 2023, after 9 months of shading treatment. Any organ not recorded during the first campaign was thus considered to have developed under shading treatments. The architectural, geometrical and experimental variables used in this study are detailed in Table 2.

**Table 2.** List of response and independent variables (left column), and data/variable types and measurement scales (right column), recorded in the experiment and used in modelling of formation and activity of flower cushions.

Response variables		
Presence of flower cushion		Boolean, ‘True/False’
Average duration of flowering episodes		Continuous (Nb censuses)
Frequency of flowering observations		Continuous [0;1]
Synchrony within GU (SI_GU)		Continuous [0;1]
Synchrony within axis (SI_Axis)		Continuous [0;1]
Synchrony within branch (SI_Branch)		Continuous [0;1]
Independent variables		
Experimental variables	Shade	Categorical, ‘Medium’ or ‘Heavy’
	Genotype	Categorical, ‘V’ or ‘B’
	Branch identity	Categorical, 9 levels
	
Architectural variables (topology)	Branching order of axis	Ordinal (2, 3, >3)
	Rank of GU (from tip)	Ordinal (1, 2, 3, 4, 5, >5)
	Position on GU (phytomer)	Ordinal (Low, Mid-low, Mid-top, Top)
	Number of GU on axis	Discrete (1–11)
	Number of nodes on the GU	Discrete (2–35)
	
Architectural and status variables of parent axis or GU	Severed axis	Boolean, True/False
	Branched GU	Boolean, True/False
	Number of flower cushions on axis	Discrete (0–57)
	Number of flower cushions on GU	Discrete (0–15)
	
Status variables of phytomers	Node type	Categorical, ‘Cataphyll’ or ‘Leaf’
	Branched node	Boolean, True/False
	Wilted vegetative bud	Boolean, True/False
	
Status variables of flower cushions	Date of emergence (category)	Ordinal, (Q1, Q2, Q3, Q4)
	Number of flowering episodes	Discrete (1–7)
	
Geometrical variables	Base diameter of GU	Continuous (mm)
	Length of GU	Continuous (cm)
	Orientation (azimuth) of axis	Categorical (N-E, S-E, S-W, N-W)
	
Other	Session of first measurement	Categorical, ‘MTG_1’ or ‘MTG_2’

**Fig. 3.**
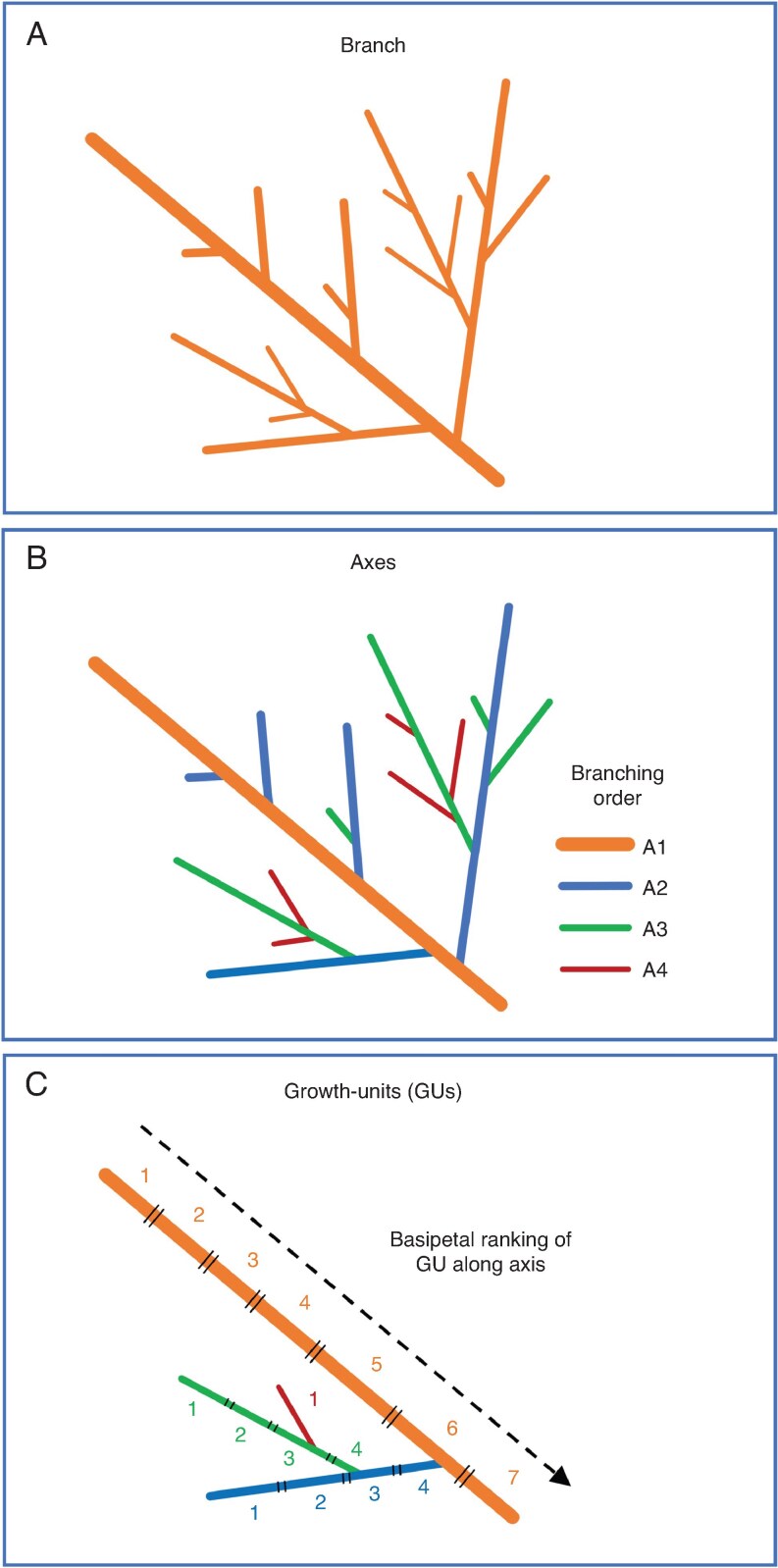
Schematic representation of topology encoding at three scales: (A) the selected branch; (B) the axis; and (C) the growth unit (GU). The selected branch comprises every component of the tree, starting from its insertion point. It is segmented into axes, each bearing a unique apical meristem. Axes are characterized by their branching order within the branch and are segmented into GUs, each corresponding to a distinct flush. GUs are characterized by their basipetal rank of succession along the axis.

**Fig. 4.**
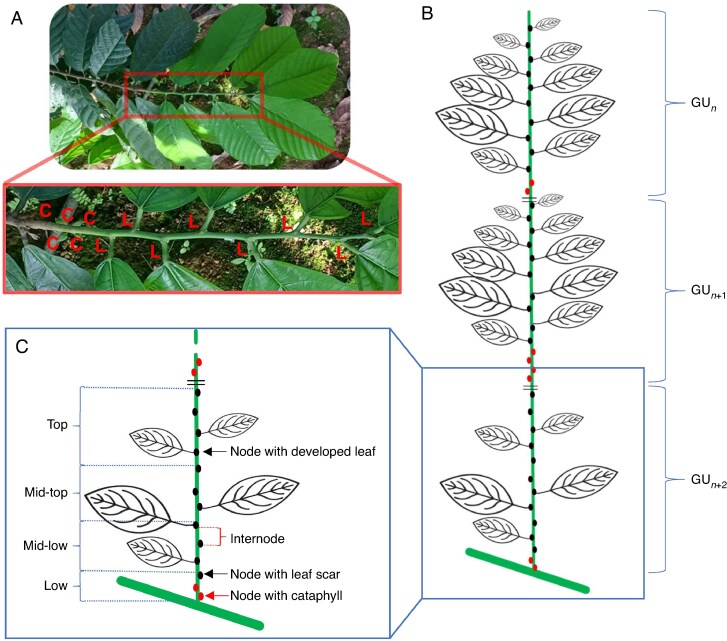
Description of cacao axis and GU structure. (A) Photograph of the distal growth unit (GU) of an axis, with the positions of nodes bearing either developed leaves ‘L’ or cataphylls ‘C’. (B) Schematic representation of a succession of GUs, nodes and developed leaves along an axis. (C) Details of a GU, comprising a succession of nodes and internodes. Each node is assigned to a quartile of position within the GU (‘low’ to ‘top’) and bears either a cataphyll (red dot) or a developed leaf (present or abscised, black dot).

For each branch of each of the nine selected trees, the architectural dataset was characterized as a collection of axes, each containing 1–10 growth units (GUs), i.e. a segment of a shoot that is produced during a single flush of growth (Figs 3 and 4B). Each GU comprised 1–28 phytomers (Fig. 4). In total, we recorded 186 axes, 557 GUs and 3857 phytomers. Axes were characterized by their branching order. Those damaged during the experiment, usually owing to insects or physical damage, resulting in a partial cut of the axis, were recorded as ‘severed axes’. Each GU was assigned a rank along its bearing axis in a basipetal manner, where rank ‘1’ indicated the youngest, most distal GU of an axis (Fig. 3).

Geometrical measurements were performed for each GU. We measured the base diameter at the midpoint of the proximal internode and recorded azimuthal orientation angles from the base to the top of the GU, which were then grouped into four quarters of azimuthal orientation. The length of the GU was measured from the base of the proximal internode to the top of the distal node.

Each phytomer was characterized by its position along the GU, divided into quartiles of node positions (Fig. 4C). A phytomer comprised an internode and a node, whose type was defined as either a cataphyll or a developed leaf (1480 and 2377 phytomers, respectively). Each node bore a principal vegetative bud, which could be dormant, branched or aborted (total of 3596, 177 and 84 phytomers, respectively), and a subordinate floral bud that could be either suppressed or developed into a flower cushion (total of 3361 and 496 phytomers, respectively). The cataphylls were consistently located in the proximal portion of the GU and had shorter internodes in comparison to nodes bearing developed leaves (Fig. 4A, C). The nodes with developed leaves remained identifiable even after leaf abscission by a characteristic large leaf scar. Because each phytomer bore a single vegetative bud, each axis was attached to a unique phytomer and characterized by its branching order.

#### Monitoring flowering activity

Following the installation of the shading treatments, we conducted a weekly monitoring from 1 April 2023 to 20 January 2024. This period encompassed 42 weekly observations, hereafter referred to as ‘censuses’. During each census, we recorded the visual observation of floral buds or flowers at anthesis on each phytomer of the branch. Notation started at the first visible appearance of floral buds at the leaf axil, corresponding to the ‘Flower bud visible’ phenological stage (BBCH 501; Niemenak *et al*., 2010). The date when floral buds were first observed on a given phytomer was designated as the date of emergence of its ‘flower cushion’. To be included as random factors, these dates of emergence were subsequently categorized into quarters corresponding to equal time periods over the entire duration of the experiment (from census 0 to 46).

To achieve the objectives of the study, the variables of interest were defined as follows:

1. Presence of a flower cushion on a phytomer. A phytomer was recorded as bearing a flowering cushion if visible flower buds and/or flowers at anthesis were observed during at least one census. A total of 496 flower cushions were recorded over the course of the study.
2. Dynamics of flowering activity on flower cushions. We defined ‘floral episodes’ as discrete periods of floral activity (presence of flower bud or flower) on a cushion, separated by rest periods of at least two consecutive censuses (i.e. ≥7 days without floral activity). Our analysis focused on two variables: (a) the average duration of completed floral episodes per cushion; and (b) the frequency of censuses during which flowering was observed. These metrics were calculated only for cushions that formed after monitoring began, excluding 40 cushions that had already developed prior to the start of observations.
3. Synchrony of flowering. To quantify the level of synchrony in flowering among flower cushions borne on similar growth units, axes or branches, we adapted the synchrony index proposed by Augspurger (1983). This index quantifies the degree of overlap in flowering periods among individuals within a population. In our study, we considered each flower cushion as an individual and calculated synchrony within populations of flower cushions borne on the same branch, axis or growth unit. The index of synchrony (*X*) for the individual *i* is given by:
  $$X_{i}=\left(\frac{1}{\left(n-1\right)}\right)\left(\frac{1}{\;f_{i}}\right)\overset{n}{\underset{\;j=1}{\sum }}\;e_{\;j\neq i}$$
  where ej is the number of censuses during which flowering of the individuals i and j overlapped, fi is the total number of censuses during which individual i was flowering, and n is the number of individuals in the population. X varies from zero, for no overlap, to one, where the flowering of a given individual perfectly overlaps with that of all individuals in the population. The average synchrony of a population (Z), consisting of n individuals, is given by:
  $$Z=\;\frac{1}{n}\;\overset{n}{\underset{i=1}{\sum }}\;X_{i}$$
  We then adapted the index to calculate a synchrony index (SI*_i_*) of each flower cushion *i*, considering the overlaps of flowering with other flower cushions at: (a) the ‘GU scale’ (*SI_GU_i_*); (b) the ‘axis scale’ (*SI_Axis_i_*), ignoring ramifications; and (c) the ‘branch scale’ (*SI_Branch_i_*), including all ramifications.
  Because our plants were young and new flower cushions formed continuously, we calculated the synchrony index for each flowering episode of each cushion only with respect to cushions that had already appeared by the end of that episode. We then averaged these episode-specific values to represent the synchrony of each flower cushion with the rest of the population. All indices were calculated for each individual flower cushion, to assess its level of synchrony with other cushions borne on similar growth unit, axis or branch. The value of the synchrony index of a flower cushion can be interpreted as the relative proportion of its flowering period that overlapped with the flowering periods of other cushions within the population.

### Data processing

Statistical analyses were conducted in R v.4.4.3 (R Core Team, 2024). We performed three sets of analyses using generalized mixed-effect linear models (GLMMs) to test the effects of various independent variables on the following: (1) the presence of flower cushions; (2) the duration of flowering episodes and the frequency of flowering censuses; and (3) the synchrony of flowering among flower cushions within GUs, axes and selected branches.

Before modelling, we filtered the datasets according to the objectives of each analysis and examined the distribution of the response variable to select the appropriate modelling method (using either ‘lme4’ or ‘glmmTMB’ packages; Bates *et al.*, 2015; Brooks *et al.*, 2024), data family, and link function for the GLMM. We identified relevant predictors and interactions based on established hypotheses. To account for the non-independence of phytomers within the same tree, we included the branch identity as a random effect in all models. Other biologically relevant sources of variation, such as branching order of the bearing axis, were considered potential confounding factors and included as random effects when they explained significant variance in the response. The details of data filtering, modelling parameters, response variables, fixed and random factors tested in each set of analyses are presented in Table 3.

**Table 3.** Details of data filtering and modelling parameters for the three sets of analyses.

	A. Probability of a phytomer bearing a flower cushion	B. Activity of flower cushions		C. Flowering synchrony
		Duration of flowering episodes	Frequency of censuses with flowering	
Filtering (data removed from analysis)	Phytomers borne on severed axes	Flower cushions with unfinished flowering episodes and/or fruit set during the experiment	Flower cushions with unfinished flowering episodes, fruit set during the experiment or single flowering episode	Two trees with very low numbers of flower cushions; GUs and axes bearing a single flower cushion
Dataset size	Phytomers *n* = 3494 Flower cushions *n* = 436	Flower cushions *n* = 316	Flower cushions *n* = 215	*n* flower cushions = 476 (SI_Branch); 468 (SI_Axis) and 451 (SI_GU)
Response variable	Presence of flower cushion (Boolean)	Average duration of flowering episodes (Continuous)	Frequency of censuses with flowering (Continuous [0;1])	Synchrony Index at GU, Axis or Branch scale (Continuous [0;1])
Modelling function	glmer (‘lme4’ package) family = binomial	glmer (‘lme4’ package) family = gamma	glmmTMB (‘glmmTMB’ package) family = beta_family	glmmTMB (‘glmmTMB’ package) family = beta_family
Fixed factors	Shade	Shade	Shade	Shade
	Genotype	Genotype	Genotype	Genotype
	Rank of GU (from tip)	Severed axis	Severed axis	Severed axis
	Position of phytomer on GU	Position of phytomer on GU	Position of phytomer on GU	Position of phytomer on GU
	Branched GU	Number of nodes on the GU	Number of nodes on the GU	Number of nodes on the GU
	Base diameter of GU	Number of flower cushions on GU	Number of flower cushions on GU	Rank of GU
	Length of GU	Length of GU	Length of GU	Length of GU
	Orientation (azimuth) of axis	Orientation (azimuth) of axis	Orientation (azimuth) of axis	Orientation (Azimuth) of axis
	Node type	Node type	Node type	Node type
	Branched node			Branching order of axis
	Wilted vegetative bud			
Random factors	Branch identity	Branch identity	Branch identity	Branch Id
	Session of first measurement	Branching order of axis	Branching order of axis	Number of flower cushions on GU
	Branching order of axis	Date of emergence (category)	Date of emergence (category)	Number of flower cushions on axis
	Number of GU on axis	Number of GU on axis	Number of GU on axis	Number of flowering episodes
				Number of GU on axis

A stepwise modelling approach was adopted, using the ‘dredge’ function from the ‘MuMIn’ package in R (Bartoń, 2024). We assessed model performance using the Akaike information criterion, checked the correlations among variables, and confirmed the absence of multicollinearity in the best-fitting models via variance inflation factors. Final model selection was based on Akaike information criterion values and biological relevance.

## RESULTS

This section presents the main results from the analyses of distributions and modelling of the studied variables. Details of structure and output of best-fitting models obtained for each analysed variable are presented in the Supplementary Data Appendix.

### Modelling the effects of architectural and experimental factors on the presence of a flower cushion on a phytomer

After filtering, the dataset included 3494 phytomers, 436 of which bore flower cushions. Table 4 presents the predictors retained in the best-fitting model, together with their associated coefficients and performance metrics (for detailed outputs, see Supplementary Data Appendix A).

**Table 4.** Effects of fixed factors in best-fitting model for the probability of flower cushion presence on a phytomer. Pr(>|*z*|): *P*-value. Significance levels: ***P < 0.001; **P < 0.01; *P < 0.05.

Presence of flower cushion	Effect (predictor and ‘modality’)	Estimate	s.e.	*z*-value	Pr (>|z|)	
	(Intercept)	−5.24	1.05	−5.00	5.73 × 10^−7^	***
Binomial (logit)	Rank of GU (from tip) ‘2’	2.22	0.34	6.53	6.81 × 10^−11^	***
*n* = 3494 phytomers (436 flower cushions)	Rank of GU (from tip) ‘3’	3.46	0.39	8.91	<2 × 10^−16^	***
	Rank of GU (from tip) ‘4’	4.37	0.42	10.33	<2 × 10^−16^	***
	Rank of GU (from tip) ‘5’	5.02	0.46	10.83	<2 × 10^−16^	***
	Rank of GU (from tip) ‘>5’	5.61	0.56	10.01	<2 × 10^−16^	***
	Position on GU ‘Mid-low’	−1.38	0.36	−3.82	0.000134	***
	Position on GU ‘Mid-top’	−1.75	0.41	−4.31	1.61 × 10^−5^	***
	Position on GU ‘Top’	−2.31	0.41	−5.64	1.72 × 10^−8^	***
	Orientation of the axis ‘S-E’	−1.22	0.22	−5.43	5.68 × 10^−8^	***
	Orientation of the axis ‘S-W’	−0.44	0.31	−1.42	0.155562	—
	Orientation of the axis ‘N-W’	−0.69	0.30	−2.27	0.022945	*
	Vegetative bud status ‘Branched’	−1.82	0.32	−5.71	1.14 × 10^−8^	***
	Base diameter of GU X Position on GU ‘Low’	0.02	0.03	0.56	0.577055	—
	Base diameter of GU X Position on GU ‘Mid-low’	0.11	0.03	3.40	0.000665	***
	Base diameter of GU X Position on GU ‘Mid-top’	0.11	0.03	3.31	0.00095	***
	Base diameter of GU X Position on GU ‘Top’	0.12	0.03	3.80	0.000145	***
	Shade ‘Heavy’	−2.04	0.59	−3.47	0.000519	***
	Genotype ‘V’	2.00	0.60	3.35	0.000813	***
	Node type ‘Leaf’	0.57	0.21	2.67	0.007709	**

The rank of the GU along the axis had the strongest effect on the probability of flower cushion presence, increasing in a basipetal (i.e. tip to base) direction along the axis. Likewise, within each GU, the likelihood of flower cushion presence increased from distal to proximal phytomers. However, this trend was modulated by an interaction with the base diameter of the GU, which became increasingly influential in promoting cushion formation at more distal phytomers. Genotype ‘V’ showed a higher probability of flower cushion presence on a phytomer. Axes oriented towards the inter-rows (North-West and South-East) had a lower probability of flower cushion presence than those oriented towards rows (North-East and South-East).

**Fig. 5.**
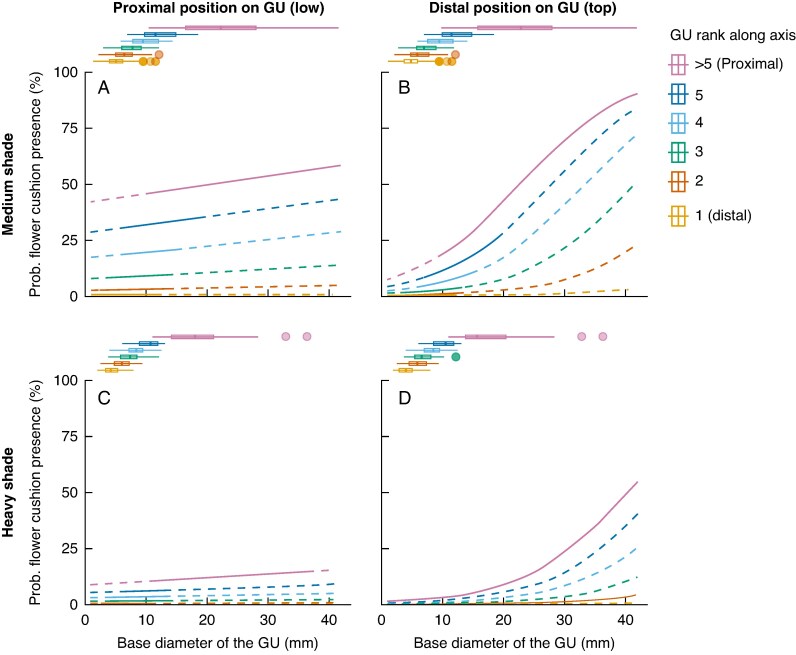
Combined effects of the base diameter (*x*-axis) and rank along the axis of a growth unit (GU, coloured lines) on probabilities of flower cushion presence, for phytomers positioned on proximal (A, C) and distal (B, D) positions on the GU, and trees subjected to medium (A, B) or heavy (C, D) shade, as predicted by the model of best fit. The calculations assumed that the phytomer bore a node with a developed leaf, had no axillary branching, and was located on an axis oriented southwest, i.e. the most common conditions in our dataset. Horizontal boxplots show distributions of measured diameter values, for each level of GU rank along the axis. Plain lines represent the predicted probabilities of presence of a flower cushion on a phytomer, calculated on values of diameter ranging within the interval of values measured in our study, for each level of GU rank along axis. Dashed lines represent the predicted probabilities before and beyond the intervals of diameter values measured in the study.

To illustrate the combined effects of the architectural and geometrical traits of a phytomer on flower cushion formation, we used the best-fitting model to calculate the probabilities of flower cushion presence (Fig. 5). The calculations assumed that the phytomer bore a node with a developed leaf, had no axillary branching, and was located on an axis oriented southwest, i.e. the most common conditions in our dataset. For phytomers on the most distal GUs of an axis (rank 1 or 2), the probability of flower cushion presence remained <5 %, regardless of shade level. Within the proximal portion of the GUs (‘lower’ GU phytomers; Fig. 4), the probability increased with the basipetal rank along the axis of the GU, and base diameter exerted only a small effect (Fig. 5A, C). The maximum probability of flower cushion presence in these proximal positions in the GU was 58 %.

In contrast, phytomers in the distal portion of the GU (Fig. 5B, D) were strongly affected by both GU rank and base diameter. When the base diameter was small (<1 cm), the probability of flower cushion presence was low (<20 %), irrespective of the rank. However, the probability increased sharply with larger diameters. Under medium shade, the relationship between base diameter and flowering probability followed a sigmoidal curve (Fig. 5B). Consequently, on GUs with small diameters, most flower cushions formed in the proximal part of the GU, with the probability of flower cushion presence increasing with the GU rank. As GU diameter increases, flower cushions develop on more distal phytomers of the GU. Notably, on GUs with large diameters (>25 mm) under medium shade, phytomers in distal positions (Fig. 5B) had a higher probability (≤90 %) of bearing flower cushions than those in proximal positions (Fig. 5A). Finally, the probability of flower cushion presence was consistently lower under heavy shade in comparison to medium shade, irrespective of the phytomer topology or geometry (Fig. 5C, D).

### Analysing the effects of architectural and experimental factors on the activity of flower cushions

**Fig. 6.**
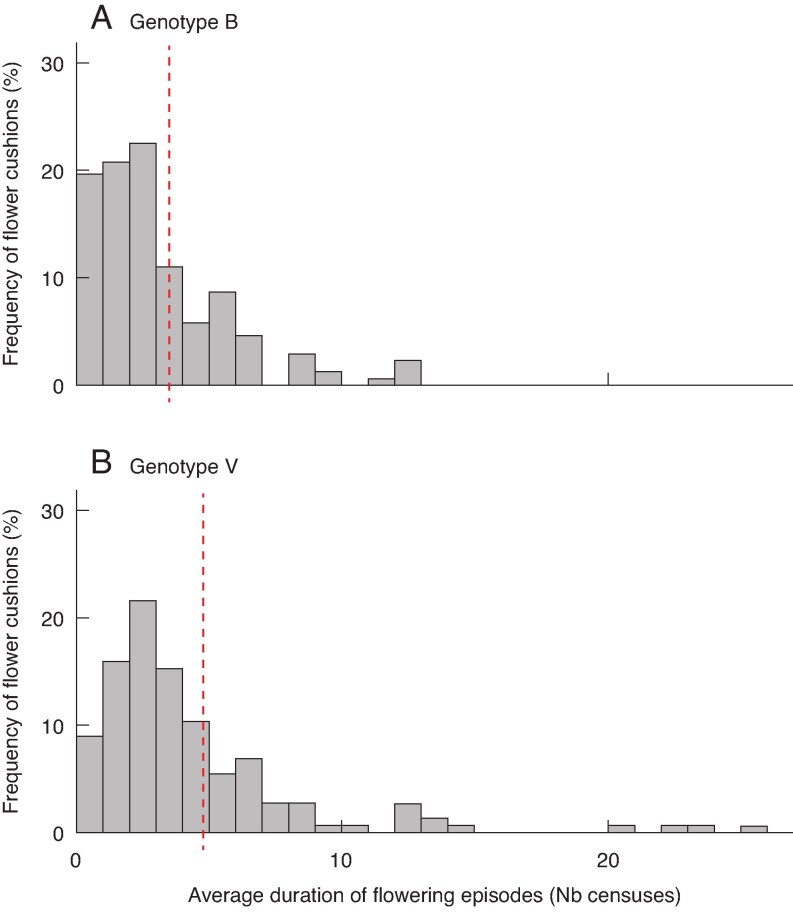
Distribution of flower cushions (as a percentage) in classes of average duration (number of consecutive censuses) of flowering episodes for genotype B (A) and genotype V (B). Red lines show the average values for each genotype.

**Fig. 7.**
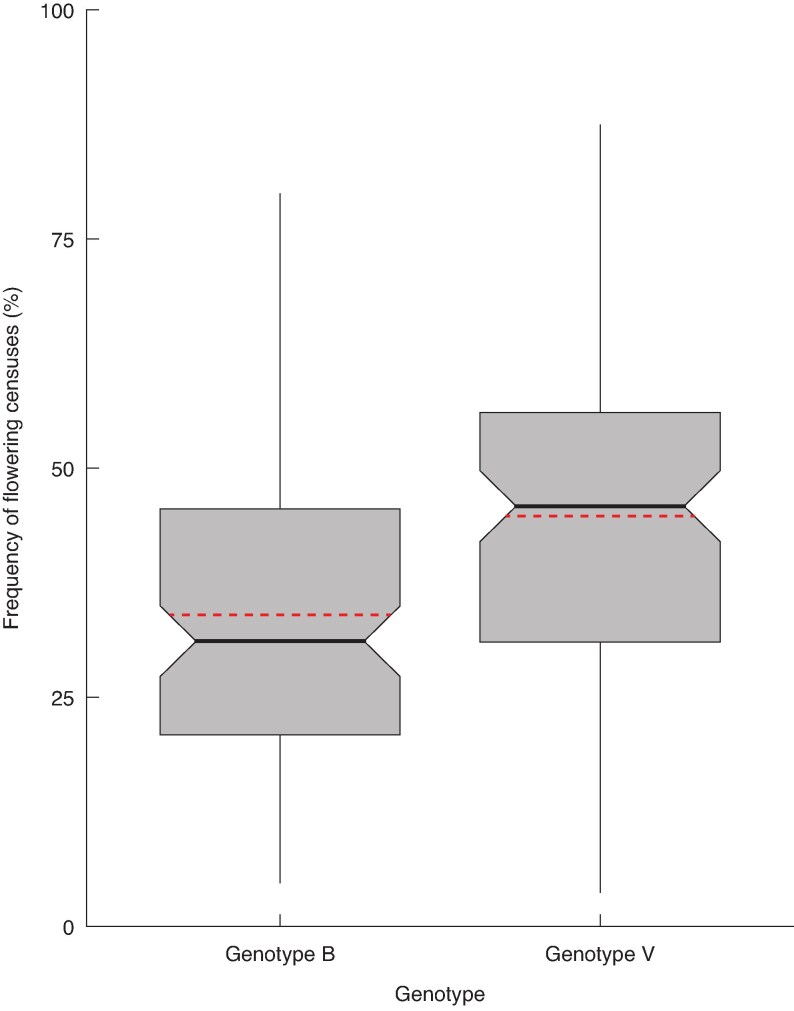
Frequencies of censuses with flowering observations, for flower cushions of genotypes B and V (medians and interquartile intervals). Red dashed lines show the average frequencies for each genotype.

Half of the flower cushions recorded during the experiment were formed before week 31 (27 October). On average, a flower cushion experienced 1.8 flowering episodes over the duration of our experiment, and the maximum number of flowering episodes observed on a single flower cushion was seven. Once they formed, flower cushions were active during 39.4 % of the censuses on average, and the average flowering episode consisted of four consecutive censuses with observations of either flower buttons or flowers at anthesis on the cushion, indicating a minimum of 21 days, considering that monitoring was performed each week. The maximum duration of a flowering episode recorded on a flower cushion was 26 consecutive censuses, or ≥167 days consecutively (Figs 6 and 7).

#### Average duration of flowering episodes

The stepwise modelling method was performed on 316 flower cushions, after filtering data. The distribution of values of average duration of flowering episode was positively skewed (Fig. 6). The model with best fit (see Supplementary Data Appendix B) yielded no significant effect of architectural factors, although flower cushions located on the severed axis had significantly shorter flowering episodes (*P* < 0.01). A positive coefficient (0.34) for genotype V indicates longer flowering episodes compared with genotype B, with average durations of 3.5 and 4.8 censuses, respectively (Fig. 6). The orientation of the GU bearing the flower cushion also had significant effect, with significantly longer flowering episodes experienced by flower cushions borne on GUs oriented towards the south-west (*P* < 0.01).

#### Frequency of flowering censuses

The stepwise modelling method was performed on 215 flower cushions, after filtering data. The model with best fit (see Supplementary Data Appendix C) yielded no significant effect of architectural and geometrical factors. Only genotype had a significant effect on the frequency of censuses with flowering activity on the flower cushions (*P* < 0.001). The positive coefficient (0.435) associated with genotype V indicated that the flower cushions on trees of this genotype were flowering more often than flower cushions of genotype B. On average, flower cushions were active during 34 and 45 % of censuses for genotype B and V, respectively (Fig. 7).

#### Flowering synchrony of flower cushions on GU, axis and tree

For each of the 476 flower cushions present on the seven selected branches used for this analysis, we used the modified synchrony index (SI) to evaluate its level of flowering overlap with other flower cushions present on the same GU, axis or branch.

**Fig. 8.**
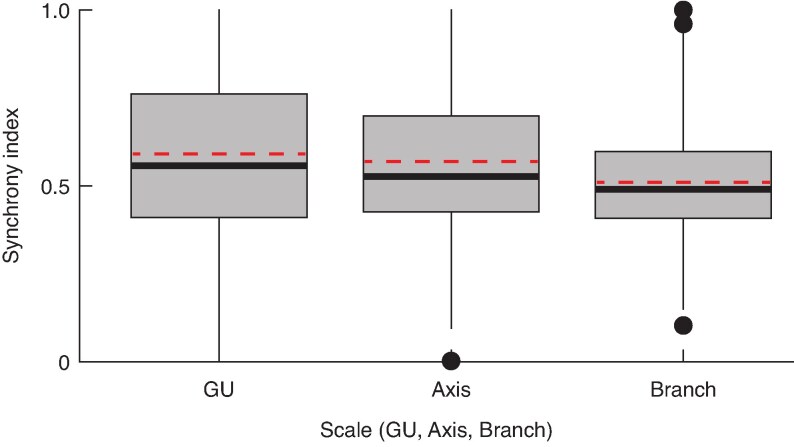
Distributions of ‘synchrony index’ values for individual flower cushions, calculated at the scale of the growth unit (GU), axis and branch (medians and interquartile intervals). Red lines indicate the average synchrony index at each scale.

On average, the flowering period of a flower cushion overlapped with 58.9, 56.7 and 50.9 % of the flowering periods of other flower cushions within the same GU, axis or branch, respectively (Fig. 8). Although the average synchrony of flowering activity between flower cushions was moderate, SI values showed wide variation, particularly at GU and axis scales. This dispersion included extreme cases of perfect flowering synchrony and asynchrony (Fig. 8). Because SI is sensitive to the number of cushions in the population (i.e. GU, axis or branch), the average SI values and their variability generally decreased with increasing scale, thereby decreasing the frequency of extreme values (close to zero or one; Fig. 8).

**Fig. 9.**
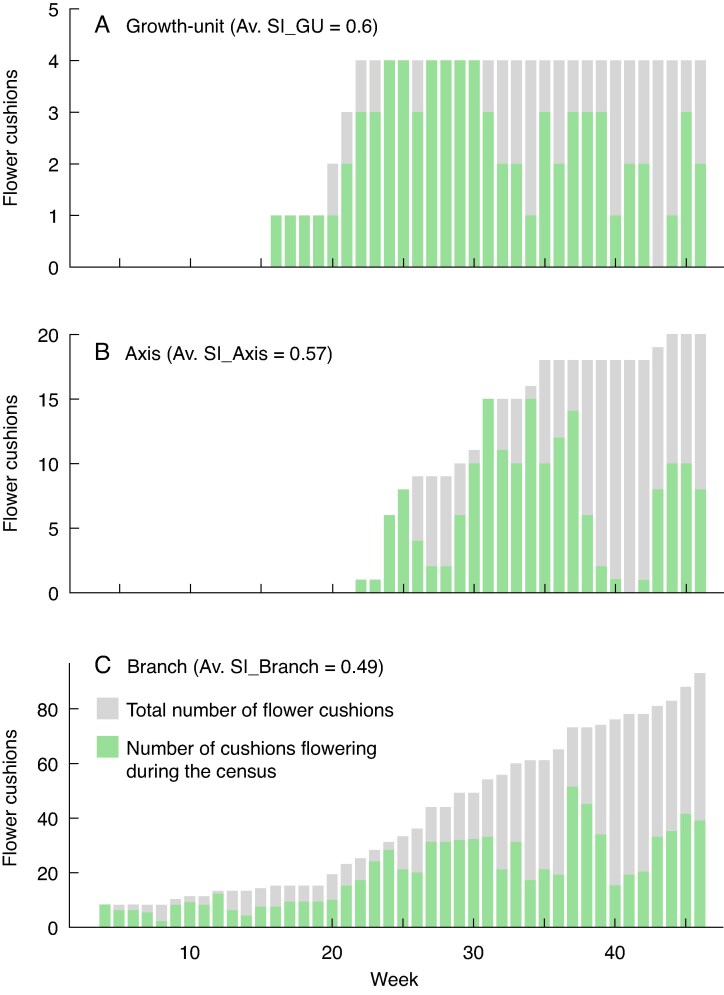
Examples of flowering overlaps between flower cushions observed on: (A) a growth unit (GU); (B) an axis; and (C) a branch, illustrating the moderate average synchrony levels recorded at each scale. Each bar represents the weekly count of developed flower cushions (grey bars) and those actively flowering (green bars) during each census.

When averaged, SI values of flower cushions borne on the same GU, axis or branch provide an overall measure of flowering synchrony within the organ. Fig. 9 illustrates representative examples of a GU, axis or branch, each with a mean SI representative of its category (Fig. 8).

The analysis of architectural and experimental variables revealed no significant effect on synchrony at GU scale. At this scale, the variability in the SI was linked primarily to the number of flower cushions on the GU, followed by the number of flowering episodes (see Supplementary Data Appendix D). In contrast, at both the axis and branch scales, the only variable showing a significant, positive effect on flower cushion synchrony was the presence of a branch on the node (SI_Axis: *P* < 0.05, estimate = 0.377; SI_Branch: *P* < 0.01, estimate = 0.355; see Supplementary Data Appendix E and F).

## DISCUSSION

Our analyses revealed that the formation of flower cushions on a phytomer was determined by both architectural (such as position within the GU, axis and branch) and geometric (namely, the diameter of the GU) factors (Table 4; Fig. 5). In addition, heavy shade had a strong suppressive effect on flower cushion formation. However, neither architectural nor geometric factors explained the variations in the average duration of flowering episodes (Fig. 6; Supplementary Data Appendix B) nor the frequency of flowering observations in flower cushions (Fig. 7; Supplementary Data Appendix C). The moderate levels of synchrony among flower cushions within GUs, axes or branches, suggested that flower cushions alternated between periods of fairly synchronous flowering, interspersed with periods of sparse flowering (Fig. 8). The variations in synchrony observed among flower cushions were not related to architectural or geometrical factors (Supplementary Data Appendix D–F). Overall, our findings in young cacao trees suggested that although architecture and geometry determined the spatial location of flower cushions, they did not influence their temporal flowering activity.

### Architecture, geometry, genetics and exogenous factors affected the ability of a node to develop flower cushions

Our results indicated that the formation of floral cushions on phytomers is controlled primarily by the ontogenetic age of the phytomer, determined by the rank of the GU on the axis and the position of the phytomer within the GU. The youngest GUs on an axis exhibited very low probabilities of bearing flower cushions, irrespective of geometrical factors (Table 4; Fig. 5). Given that only unsevered axes were analysed, this suggested that a minimum ontogenetic age is required for flower cushion formation. This observation aligns with previous studies reporting that flowers typically develop on shoots of sufficient diameter, often where leaves have fallen, and rarely on the youngest flushes (Alvim, 1984; Mialet, 1990; Bartley, 2005). As we move downwards along the GUs on an axis, the ontogenetic age increases, as does the probability of flower cushion presence. The increase in base diameter of the GU also has a positive effect on the presence of flower cushions, in relationship to the position of the node within the GU (Fig. 5). Given that an increase in diameter is both a growth parameter and a function of time for a given GU, our findings suggest that the formation of flower cushions follows this sequence:

1. Early formation: once a GU has reached an ontogenetic age sufficient for flowering, the proximal nodes, including nodes with cataphylls, are the first to develop flower cushions.
2. Progressive development: as the diameter of the GU increases, additional nodes along the upward gradient of the GU progressively acquire the capacity to develop flower cushions.
3. Definitive distribution: when the GU reaches its maximum potential for flower cushion formation, the distal phytomers of a GU eventually exhibit higher probabilities of bearing flower cushions than the proximal phytomers.

There were no notable differences between axes in terms of reproductive capacity (Table 4), and every studied axis followed a similar developmental pattern: youngest GUs supported photosynthetically active leaves, and older GUs transitioned from vegetative to reproductive states, with cushions gradually forming along the acropetal gradient of nodes on the GU. This contrasts with other temperate and tropical fruit trees, where vegetative and reproductive axes often differ geometrically. In apple, for example, variations in the proportion of leaf and axis biomass reflect functional differentiation (the ‘axialization’ of botanical entities; Lauri and Térouanne, 1998; Lauri and Kelner, 2001; Normand *et al.*, 2009; Lauri and Normand, 2017). However, functional differentiation in vegetative and reproductive fate can occur between orthotropic stems, primary plagiotropic axes of the jorquette, and higher-order plagiotropic branches on cacao trees grown from seeds, as reported by Alvim (1984) and Bartley (2005). Additionally, our study examined only one branch per tree, representing an average of 23 % of the canopy of the tree, which might not fully reflect tree-wide trends.

Architectural and geometrical traits strongly influence the ability of a node to develop flower cushions, but genotype and shading also play significant roles (Table 4). Probabilities of flower cushion presence on nodes borne on GUs with similar diameter and ontogenetic age were higher in genotype V than in genotype B. Differences in the intensity, timing and temporal distribution of flowering between cacao genotypes are well documented, particularly among populations of contrasting geographical origins (Boyer, 1970; Mossu *et al.*, 1981; Bartley, 2005). But our observations suggest that genetic factors could partially control both the ability of the tree to establish flower cushions and the dynamics of their flowering activity. These findings open new opportunities for future studies on the genetic traits associated with the precocity and abundance of flower cushion establishment in cacao, in addition to the underlying physiological and molecular mechanisms, with potential applications for varietal selection. In both genotypes, however, trees under heavy shade exhibited much lower frequencies of flower cushion formation than those under medium shade (Fig. 5), consistent with prior research (Alvim, 1966; Boyer, 1970; Asomaning *et al.*, 1971; Ampofo and Bonaparte, 1979; Ahenkorah *et al.*, 1987; Almeida and Valle, 2007; Famuwagun and Agele, 2021). In our study, trees under heavy shade received in average only 10 % of ambient PAR, and nodes in similar topological positions and with comparable vegetative development had considerably reduced probability of bearing flower cushions compared with those under medium shade. This suggests that heavy shade strongly restricts the ability of a phytomer to develop a flower cushion.

### Once initiated, the activity of flower cushions appeared unrelated to architectural and geometrical factors, at least during early flowering

The flowering activity observed in our experiment consisted of relatively short flowering episodes, 4 weeks on average, interspersed with similar rest intervals (Fig. 6). Trees of genotype V exhibited both longer flowering episodes and more frequent flowering compared with genotype B, indicating greater overall flowering activity of their flower cushions (Figs 6 and 7). However, the high frequency of single-census episodes, which were more frequently observed for genotype B, suggests that floral bud abortion was common for this genotype (Fig. 6). This increased rate of short, aborted flowering episodes contributes to the low average duration of recorded flowering episodes on genotype B. In addition, given that we did not count the number of flowers produced per episode, it remains possible that genotype B, despite its shorter flowering episodes, might have produced a higher number of flowers per episode.

The analysis of flowering synchrony at the GU, axis and branch scales offered insights into the temporal variability of flowering across these scales. At each scale, the moderate average synchrony values (Fig. 8) indicate that cushions periodically flower in near-synchrony, interspersed with intervals in which only some flower cushions are active while others remain quiescent (Fig. 9). This pattern might explain the impression of continuous flowering reported in previous studies (Alvim, 1966, 1984; Mossu *et al.*, 1981; Bartley, 2005).

The lack of associations between architectural or geometrical factors and flowering activity in this study (Supplementary Data Appendix B–F) is likely to reflect the unstructured, continuous flowering typical of young cacao trees during their early flowering seasons, as reported by Alvim (1966) and Sale (1969). It is expected that more structured cycles, with more synchronous flushing and flowering rhythms, would emerge later in the life of the tree, influenced by climate variations and the rhythmic alternation between vegetative growth, flowering and fruiting (Boyer, 1970; Alvim, 1984; Young, 1984). Further research on mature trees with established phenological rhythms is needed to gain a better understanding of the patterns of flower cushion activity, levels of synchrony, and the factors driving these variations.

### On the need to explore the internal mechanisms regulating the formation of flower cushions

Our findings show that the reproductive fate of a phytomer varies over time and in response to the growth status of its bearing GU, thereby raising questions about the mechanisms controlling the formation of flower cushions in cacao and other cauliflorous species. A key observation is that the delayed activation of subordinate (floral) buds in cacao appears more akin to ‘proleptic’ bud break than to classical floral bud dormancy.

In cacao, subordinate meristems form during primary growth of an axis but can remain suppressed for extended periods (Greathouse and Laetsch, 1969; Wheat, 1979; Swanson *et al.*, 2008). In seedling-grown cacao, the tree typically acquires reproductive ability only after the first crown of plagiotropic branches develops, usually after 2–3 years of growth. This event triggers the formation of floral cushions on the oldest nodes of the plant, demonstrating the capacity of floral buds to remain suppressed for prolonged periods (Alvim, 1984; Wood and Lass, 2001). It also underscores the role of an ontogenetic event in controlling the activation of these long-suppressed floral buds. The phenomenon of prolonged suppression and delayed activation of reproductive meristems appears somewhat specific to cauliflorous species, but the endogenous mechanisms involved have not yet been studied. We can only speculate on these mechanisms based on the existing knowledge of proleptic bud break and floral bud dormancy.

In our study, the trees were grafted from budwood collected from plagiotropic axes of adult trees that had already produced flowers and fruits. In this context, the consistent relationships observed between ontogenetic age, bearing GU diameter and the probability of flower cushion formation suggest that internal factors also regulate the activation of floral buds on plagiotropic axes. In cases of proleptic branching or delayed floral bud break, which are commonly observed in tropical perennials in favourable environmental conditions, dormancy and bud break are generally assumed to be regulated remotely by certain organs, such as apical meristems, young leaves, flowers or fruits. This form of growth inhibition, known as para-dormancy, operates within the tree and is mediated by hormonal signals and source–sink relationships between organs (Nozeran *et al.*, 1971; Lang *et al.*, 1987; Shimizu-Sato and Mori, 2001; Ongaro and Leyser, 2007; Smith and Samach, 2013; Capelli *et al.*, 2021). Many studies on the physiology of flowering in cacao have focused on endogenous mechanisms related to fruit set, particularly sexual self-compatibility and floral abscission (Aneja *et al.*, 1999; Hasenstein and Zavada, 2001; Almeida and Valle, 2007; Swanson *et al.*, 2008). However, there are no reports addressing the physiological factors involved in floral induction and floral bud activation in cacao. A hormonal control of floral induction has been hypothesized based on girdling experiments, which demonstrated that the removal of a ring of bark stimulated flowering above the wound while suppressing it below (Alvim, 1984). However, it remains unclear whether these variations in flowering were attributable to changes in the activity of established flower cushions or the development of new ones, because neither hormonal nor carbohydrate balances were assessed in this or any previous study.

Our results also indicate that floral bud activation was not synchronous within GUs but instead depended on the position of the node within the GU (Table 4; Fig. 5). This suggests that localized control mechanisms might maintain dormancy in subordinate buds at distal positions on the GU. The axes examined in our study were intact and bore no fruit, meaning that potential sources of localized inhibition on floral bud activation might have included leaves, other flower cushions or active axillary buds or shoots. Our results showed that the presence of a branched shoot at a node had a significant negative effect on flower cushion formation, whereas the loss of the principal axillary bud (aborted or cut out) had no significant effect (Table 4). The differences in the timing of flower cushion formation across nodes within a GU might also be linked to the presence and/or photosynthetic activity of the leaf attached to the node. Unfortunately, we were unable to monitor leaf abscission dates or measure all leaves that had abscised before the architectural measurement campaigns. Future research should investigate this hypothesis by exploring the potential inhibitory effects of attached leaves and examining relationships between flower cushion formation, leaf abscission timing and carbohydrate balance at the node, GU and axis levels.

### Conclusion

Our study examined the effects of architecture and growth on the presence and activity of flower cushions in cacao, a cauliflorous tropical fruit tree. Architectural measurements and weekly monitoring of flowering activity at the node level were conducted on single branches of cacao trees from two genotypes, grown under either 60 % (medium shade) or 10 % (heavy shade) ambient sunlight. Inactive floral buds developed into flower cushions only when the phytomer reached a certain ontogenetic age. Beyond this threshold, the probability of flower cushion formation depended on the position of the node within the growth unit and its diameter. Although flower bud activation was under endogenous control, heavy shade had a strong negative impact on flowering, regardless of architectural and growth characteristics. Temporal activity of the flower cushions was not related to architectural or growth traits of the phytomer, and flowering occurred in moderate synchrony within the growth unit, axis and branch. These findings provide new insights into the factors regulating floral bud outgrowth and the patterns and variations of flowering activity in the persistent flower cushions of cacao. Future investigations should focus on adult trees with well-established flower cushions to gain a more comprehensive understanding of flower cushion activity and the effect of fruiting on the functioning of flower cushions. A deeper understanding of the factors involved in the variations of flowering activity of flower cushions, in addition to their variation within a plant, would be valuable for agronomic and breeding purposes.

## Supplementary Material

mcaf107_Supplementary_Data

## SUPPLEMENTARY DATA

Supplementary data are available at *Annals of Botany* online and consist of the following. Appendix: Detailed model structures and outputs for the analysis of architectural and geometrical effects on flowering parameters.
