# Supplementary material for: A spatial perspective on flowering in cauliflorous cacao: architecture defines flower cushion location, not its early activity
Source: Ann Bot. 2025 Jun 10;136(2):309–23. doi: 10.1093/aob/mcaf107 (PMC12445857; doi:10.1093/aob/mcaf107)
Supplement: mcaf107_Supplementary_Data [file mcaf107_supplementary_data.zip › Appendix Models Outputs.pdf]

Appendix: Detailed model structures and outputs for the analysis of architectural and geometrical effects on flowering parameters

A. “Probability of a phytomer bearing a flower cushion”

A.1. Model Formula:

Presence of flower cushion ~ Rank of GU (from tip) + Position on GU + (Position on GU : Base diameter of GU) + Shade + Orientation of axis + Branched node + Node Type + Genotype + (1 | Branch Id) + (1 | Branching order of axis) + (1 | Number of GU on axis) + (1 | Session of 1st measurement)

A.2. Family and Link Function:

Binomial (link = logit)

A.3. Random Effects Structure:

(1 | Branch Id) + (1 | Branching order of axis) + (1 | Number of GU on axis) + (1 | Session of 1st measurement)

A.4. Fixed Effects Estimates

term	estimate	std.error	statistic	p.value	conf.low	conf.high
(Intercept)	-5.244	1.049	-5.000	**<0.001**	-7.300	-3.188
Rank of GU (2)	2.218	0.340	6.525	**<0.001**	1.552	2.884
Rank of GU (3)	3.457	0.388	8.907	**<0.001**	2.696	4.218
Rank of GU (4)	4.370	0.423	10.332	**<0.001**	3.541	5.199
Rank of GU (5)	5.021	0.464	10.825	**<0.001**	4.112	5.930
Rank of GU (5)	5.610	0.561	10.005	**<0.001**	4.511	6.710
Position on GU (mid-low)	-1.380	0.361	-3.819	**<0.001**	-2.088	-0.672
Position on GU (mid-top)	-1.754	0.407	-4.313	**<0.001**	-2.552	-0.957
Position on GU (top)	-2.311	0.410	-5.638	**<0.001**	-3.114	-1.507
Shade (Heavy)	-2.043	0.589	-3.471	**0.001**	-3.197	-0.889
Orientation of axis (SE)	-1.219	0.225	-5.428	**<0.001**	-1.660	-0.779
Orientation of axis (SW)	-0.439	0.309	-1.420	0.156	-1.046	0.167
Orientation of axis (NW)	-0.689	0.303	-2.274	**0.023**	-1.282	-0.095
Branched node (TRUE)	-1.818	0.318	-5.708	**<0.001**	-2.442	-1.193
Node Type (Leaf)	0.566	0.213	2.665	**0.008**	0.150	0.983
Genotype (V)	1.999	0.597	3.348	**0.001**	0.829	3.169
Position on GU (low) : Base diameter of GU	0.018	0.032	0.558	0.577	-0.044	0.079

term	estimate	std.error	statistic	p.value	conf.low	conf.high
Position on GU (mid-low): Base diameter of GU	0.111	0.033	3.404	**0.001**	0.047	0.176
Position on GU (mid-top) : Base diameter of GU	0.108	0.033	3.305	**0.001**	0.044	0.172
Position on GU (top) : Base diameter of GU	0.118	0.031	3.799	**<0.001**	0.057	0.178

A.5. Random Effects Estimates

effect	group	term	estimate
ran_pars	Number of GU on axis	sd__(Intercept)	0.957
ran_pars	Branch Id	sd__(Intercept)	0.776
ran_pars	Branching order of axis	sd__(Intercept)	1.019
ran_pars	Session of 1st measurement	sd__(Intercept)	0.572

A.6. Model Performance

AIC	AICc	BIC	R2_conditional	R2_marginal	ICC	RMSE	Sigma
1,597.2	1,597.6	1,745.1	0.740	0.511	0.467	0.256	1

B. “Average duration of flowering episodes”

B.1. Model Formula:

Av. duration of flowering episodes ~ Orientation of axis+ Node Type + Severed axis + Genotype + (1 | Date of emergence ‘category’) + (1 | Number of GU on axis)

B.2. Family and Link Function:

Gamma (link = log)

B.3. Random Effects Structure:

(1 | Date of emergence ‘category’) + (1 | Number of GU on axis)

B.4. Fixed Effects Estimates

Term	estimate	std.error	statistic	p.value	conf.low	conf.high
(Intercept)	1.159	0.232	4.975	**<0.001**	0.702	1.615
Orientation of axis (SE)	-0.09	0.109	-0.89	0.373	-0.31	0.116
Orientation of axis (SW)	0.386	0.133	2.892	**0.003**	0.124	0.648
Orientation of axis (NW)	0.114	0.144	0.791	0.428	-0.16	0.397
Node Type (Leaf)	0.109	0.073	1.481	0.138	-0.03	0.254
Severed axis (TRUE)	-0.40	0.141	-2.86	**0.004**	-0.68	-0.12
Genotype (V)	0.340	0.096	3.533	**<0.001**	0.151	0.528

B.5. Random Effects Estimates

Group	term	estimate
Number of GU on axis	sd__(Intercept)	0.205
Date of emergence ‘category’	sd__(Intercept)	0.260
Residual	sd__Observation	0.682

B.6. Model Performance

AIC	AICc	BIC	R2_conditional	R2_marginal	ICC	RMSE	Sigma
1,378	1,379	1,416	0.292	0.088	0.223	3.167	0.682

C. “Frequency of censuses with flowering”

C.1. Model Formula:

Frequency of censuses with flowering ~ Genotype + (1 | Date of emergence ‘category’) + (1 | +Number of GU on axis)

C.2. Family and Link Function:

Beta family

C.3. Random Effects Structure:

(1 | Date of emergence ‘category’) + (1 | Number of GU on axis)

C.4. Fixed Effects Estimates

term	estimate	std.error	statistic	p.value	conf.low	conf.high
(Intercept)	-0.638	0.084	-7.585	**<0.001**	-0.803	-0.473
Genotype (V)	0.435	0.10	4.205	**<0.001**	0.232	0.637

C.5. Random Effects Estimates

Group	term	estimate
Date of emergence ‘category’	sd__(Intercept)	0.064
Number of GU on axis	sd__(Intercept)	0.066

C.6. Model Performance

AIC	AICc	BIC	R2_conditional	R2_marginal	ICC	RMSE	Sigma
-152.5	-152.2	-135.7	0.250	0.212	0.0478	0.169	7.217

D. Flowering synchrony at GU scale

D.1. Model Formula:

Synchrony Index (GU) ~ (1 | Branch Id) + (1 | Branching order of axis) + (1 | Number of flower cushions on GU) + (1 | Number of flowering episodes)

D.2. Family and Link Function:

Beta family

D.3. Random Effects Structure:

(1 | Branch Id) + (1 | Branching order of axis) + (1 | Number of flower cushions on GU) + (1 | Number of flowering episodes)

D.4. Fixed Effects Estimates

term	estimate	std.error	statistic	p.value	conf.low	conf.high
(Intercept)	0.276	0.234	1.182	0.237	-0.182	0.734

D.5. Random Effects Estimates

Group	term	estimate
Branch Id	sd__(Intercept)	0.238
Branching order of axis	sd__(Intercept)	0.118
Number of flower cushions on GU	sd__(Intercept)	0.354
Number of flowering episodes	sd__(Intercept)	0.242

D.6. Model Performance

AIC	AICc	BIC	R2_conditional	R2_marginal	ICC	RMSE	Sigma
-229.1	-228.9	-204.7	0.499	0.00	0.500	0.240	1.609

E. Flowering synchrony at Axis scale

E.1. Model Formula:

Synchrony Index (Axis) ~ Branched node + (1 | Branch Id) + (1 | Branching order of axis) + (1 | Number of GU on axis) + (1 | Number of flower cushions on Axis) + (1 | Number of flowering episodes)

E.2. Family and Link Function:

Beta family

E.3. Random Effects Structure:

(1 | Branch Id) + (1 | Branching order of axis) + (1 | Number of GU on axis) + (1 | Number of flower cushions on Axis) + (1 | Number of flowering episodes)

E.4. Fixed Effects Estimates

term	estimate	std.error	statistic	p.value	conf.low	conf.high
(Intercept)	0.966	0.566	1.706	0.088	-0.143	2.075
Branched node	0.377	0.183	2.059	**0.039**	0.018	0.735

E.5. Random Effects Estimates

Group	term	estimate
Branch Id	sd__(Intercept)	0.562
Branching order of axis	sd__(Intercept)	0.793
Number of GU on axis	sd__(Intercept)	0.239
Number of flower cushions on Axis	sd__(Intercept)	0.437
Number of flowering episodes	sd__(Intercept)	0.105

E.6. Model Performance

AIC	AICc	BIC	R2_conditional	R2_marginal	ICC	RMSE	Sigma
-205.1	-204.8	-172.1	0.939	0.007	0.939	0.183	3.692

F. Flowering synchrony at Branch scale

F.1. Model Formula:

Synchrony Index (Branch) ~ Branched node + (1 | Branch Id) + (1 | Number of flower cushions on Axis) + (1 | Number of flowering episodes)

F.2. Family and Link Function:

Beta family

F.3. Random Effects Structure:

(1 | Branch Id) + (1 | Number of flower cushions on Axis) + (1 | Number of flowering episodes)

F.4. Fixed Effects Estimates

term	estimate	std.error	statistic	p.value	conf.low	conf.high
(Intercept)	0.072	0.123	0.585	0.558	-0.169	0.314
Branched node	0.355	0.120	2.953	**0.003**	0.119	0.590

F.5. Random Effects Estimates

Group	term	estimate
Branch Id	sd__(Intercept)	0.278
Number of flower cushions on Axis	sd__(Intercept)	0.168
Number of flowering episodes	sd__(Intercept)	0.077

F.6. Model Performance

AIC	AICc	BIC	R2_conditional	R2_marginal	RMSE	Sigma
-436.4	-436.2	-411.5	0.607	0.037	0.594	0.134
